# Stability of Phenolic Compounds in Grape Stem Extracts

**DOI:** 10.3390/antiox9080720

**Published:** 2020-08-08

**Authors:** Irene Esparza, María José Cimminelli, Jose Antonio Moler, Nerea Jiménez-Moreno, Carmen Ancín-Azpilicueta

**Affiliations:** 1 Department of Sciences, Universidad Pública de Navarra, Campus Arrosadía s/n, 31006 Pamplona, Spain; irene.esparza@unavarra.es (I.E.); mariajose.cimminelli@unavarra.es (M.J.C.); 2 Institute for Advanced Materials (InaMat), Universidad Pública de Navarra, 31006 Pamplona, Spain; 3 Department of Statistics and Operational Research, Universidad Pública de Navarra, Campus Arrosadía s/n, 31006 Pamplona, Spain; jmoler@unavarra.es

**Keywords:** grape stem extract, stability, temperature, light, storage, phenolic compounds

## Abstract

Grape stem is rich in phenolic compounds, especially stilbenes. These antioxidants can be degraded during the storage of grape stem extracts for long periods of time. The aim of this work was to analyze the stability of Mazuelo stem extracts during storage at 25 and 40 °C, in two different light conditions (amber and transparent vials). The stability of the antioxidants was studied after 2, 4 and 6 months of conservation. Gallic acid and the quercetin derivative concentration were stable throughout the storage period. In contrast, catechin disappeared from all the extracts in just two months of storage. Anthocyanins were significantly affected by temperature, and light enhanced their degradation when the extracts were kept at 40 °C. Resveratrol and viniferin showed a similar behavior. Their concentration decreased from the beginning of storage, and in both cases, they were significantly affected by both temperature and light.

## 1. Introduction

Food processing industries generate large amounts of waste, that threaten the environment and represent a significant economic loss. This problem represents one of the main challenges to be addressed in the coming years to solve the growing environmental problems. On the other hand, in most cases, agri-food residues can be converted into valuable compounds, due to their wealth of bioactive compounds. The extraction of these compounds can be very interesting and profitable from an economic point of view. Among them, phenolic compounds constitute a relevant group of substances, due to their proven beneficial properties for health. They have been studied in depth in recent years, and are known to have a wide range of physiological properties, such as antioxidants and antimutagens, as well as having antimicrobial, anti-inflammatory and anticancer activity [1,2]. Although phenolic compounds are widely distributed in plant foods, their extraction from agri-industrial by-products is preferred [3]. For this reason, in recent years, there has been great interest in obtaining polyphenols from plant residues, suitable for applications in the pharmaceutical industry, as food additives and supplements, or in cosmetics.

In this context, the oenological industry generates many residues rich in phenolic compounds, which pose a serious economic problem and an environmental impact. The main by-products of winemaking are grape stem, grape pomace, seeds and wine lees, among others. All of them account for more than 30% of the total raw material of a winery. Grape stems are the vegetative structure that directly supports the berries, and they are eliminated at the beginning of the winemaking process, to prevent them from contributing astringency to the wine and negatively affecting the product quality. In some countries, grape stems have been used as fertilizer, as well as to obtain proteins and to produce liqueurs [4]. However, the use of grape stems to produce fertilizers is limited, because it requires prior extraction of polyphenols due to their antimicrobial activity, which could hinder the composting process [5]. Different studies have reported that grape stems are an important source of dietary fiber [5,6], but also an important source of antioxidants, highlighting *trans*-resveratrol and trans-ε-viniferin, although their concentration will vary depending on the grape vintage and the wine-growing area [7,8]. In view of their significant content in phenolic compounds, the extraction of antioxidants could be one of the most interesting ways of taking advantage of this by-product.

However, despite the high potential of phenolic compounds, the use of by-products from the agri-food industry is conditioned by their high instability. Polyphenols have unsaturated bonds and a strong antioxidant capacity, that make them sensitive to heat, pH variations, light, enzymatic activities, and the presence of metal ions and oxygen [9]. Furthermore, these compounds showed a lack of stability during long-term storage [10]. Among the factors that can affect the stability of these compounds, temperature stands out, since their molecular structures are thermolabile. For example, high temperatures can induce the epimerization of (−)-epicatechin to (−)-catechin and (+)-catechin to (+)-epicatechin [11]. Likewise, resveratrol is a compound which is quite unstable against the action of different factors such as oxygen, heat, light and pH, which can reduce or destroy its effectiveness [12,13]. Rocha-Parra et al. [14] found that the stability of some polyphenols (malvidin-3-glucoside, catechin and epicatechin) decreased as the water activity of the extract increased. Volf et al. [15] analyzed the stability of some phenolic compounds (gallic acid, catechin and vanillic acid) under UV light and temperature. These authors compared the stability of these compounds in the form of pure standards, versus the same substances in grape seed and spruce bark extracts, and found a greater degradation when they were used as pure standards. Albuquerque et al. [16] have reported that the stability results of flavan-3-ols are highly dependent on the plant matrix source. This highlights the need to know and control both the extraction processes and the stability and conservation conditions for each specific extract.

For all these reasons, the stability of phenolic compounds will depend not only on its chemical nature, but also on the overall composition of the extract, since the different compounds present in the matrix could enhance or mitigate the degradation processes of a specific compound. Grape stem extracts are rich in phenolic compounds that can be sensitive to temperature changes, undergo photolytic reactions and easily oxidize. However, in the literature search made, no work was found analyzing the stability of these antioxidants in grape stem extracts. Moreover, considering that the extraction of these compounds from grape stems is seasonal, moderate and even prolonged periods of storage will probably be required before use. Thus, it is even more necessary to determine the best storage conditions of the extracts for the antioxidant’s stability. In a previous work, we optimized the extraction method of polyphenols from Mazuelo grape stems [8]. Here, the aim was to determine the stability of these grape stems extracts over a 6-month period, when they were subjected to different storage conditions (two temperatures, 25 and 40 °C, both in the absence or in the presence of light).

## 2. Materials and Methods

### 2.1. Samples

Extracts were obtained from grape stems of Mazuelo variety from the Estación de Viticultura y Enología de Navarra, in northern Spain (2016 harvest). Prior to extraction, the stems were oven dried (Ing Climas, Barcelona, Spain) at 25 °C until constant weight. They were subsequently ground in a coffee grinder (Moulinex, Ecully, France) and passed through a 300 µm sieve. To obtain the grape stem extract, the dried and ground stem was macerated in 50% *v/v* ethanol/water (solid/solvent ratio 1:100, *w/v*), for 24 h at 40 °C. After the incubation, the extracts were centrifuged (8000 rpm for 15 min), filtered and lyophilized. The characterization of the extract thus obtained was published in a previous work [8]. To perform the stability study, 50 mg of the lyophilized extract were stored in transparent (T) and in amber vials (A), at 25 °C and 40 °C for 6 months. The samples were stored under fluorescent lamps. Nine replicates were performed for each of the conservation conditions studied, and three vials of each type were taken every two months for characterization. At the end of each storage period, the samples were kept at −20 °C for subsequent analysis. Prior to analysis, the lyophilized extract in each vial was reconstituted in 10 mL of methanol.

### 2.2. Determination of the Antioxidant Capacity of Grape Stem Extracts during Storage

The antioxidant capacity of the grape stem extracts was determined by the DPPH (2,2-diphenyl-1-picrylhydracyl) assay according to Brand-Williams et al. [17] First, a standard solution of the radical was prepared by dissolving 25 mg of DPPH in 100 mL of methanol. An aliquot of that solution was taken, and diluted to obtain a DPPH working solution, with an absorbance of 0.90 ± 0.02 at 517 nm. Next, 150 μL of the extract and 2.85 mL of the DPPH working solution were mixed. They were left in the dark for 30 min, and the absorbance at 517 nm was measured on a UV/Vis spectrophotometer (Jenway 7315, Staffordshire, UK). For the quantification, a Trolox calibration curve between 0.05 and 0.80 mM was used. The results were expressed as mmol of Trolox per gram of dry extract.

### 2.3. Total Phenolic Content Determination of Grape Stem Extracts during Storage

Total polyphenol content was determined using the Folin-Ciocalteu method [18]. Briefly, 0.1 mL of the extract were mixed with 7.9 mL of deionized water and 0.5 mL of the Folin-Ciocalteu reagent. It was left to react for 2 min, and then 1.5 mL of Na_2_CO_3_ were added. After 2 h in the dark, the absorbance of the samples was measured at 765 nm. A calibration curve with gallic acid concentrations between 0.2 and 4 mM was used for the quantification. Results were expressed as mmol of gallic acid per gram of dry extract.

### 2.4. Identification and Quantification of Phenolic Compounds in Grape Stem Extracts by HPLC

The phenolic composition of the extracts was analyzed by HPLC (Waters Div., Milford, MA, USA), using a liquid chromatograph equipped with two 515 pumps and a 717 Plus autosampler. For the identification and quantification of the compounds, a Photodiode Array 996 detector programmed for scanning from 200 to 600 nm was used. A reverse phase column (Zorbax Eclipse Plus C18, 250 × 4.6 mm, particle size 5 µm) at 30 °C was used. Chromatograph control and data processing were performed using Empower 2.0 software.

The separation of phenolic compounds was performed using the method proposed by Barros et al. [19], with slight modifications. The mobile phases used were: A (water: 85% formic acid, 99:1 *v/v*) and B (acetonitrile:85% formic acid, 99:1 *v/v*). The flow rate was 1 mL/min and a gradient program of 60 min was used (t in min; %A): (0; 95%), (15; 85%), (22; 80%), (25, 80%) (35, 70%), (45; 50%), (50, 5%), (55, 95%), (60, 95%). The injection volume was 30 μL. All the solvents used were HPLC quality from Scharlab (Barcelona, Spain).

The identification of the compounds was made by the double coincidence of the UV-visible spectrum characteristic of each compound, and of the retention time of the corresponding standard. The following phenolic compounds were identified in the extracts: gallic acid, catechin, malvidin-3-glucoside, quercetin, a quercetin derivative expressed as quercetin-3-glucoside, resveratrol, viniferin, and an unknown anthocyanin. For the quantification of these antioxidants, a calibration curve for each of them was made, with the exception of the unidentified anthocyanin, which was quantified with the calibration curve of malvidin-3-glucoside.

### 2.5. Statistical Analysis

Different statistical treatments were performed on the data. Firstly, the concentration values obtained for each of the phenolic compounds at different times were divided by their average concentration at the beginning of the experiment. When the concentration of a certain compound was below the limit of quantification (LOQ) of the technique, a left-censored data completion model called Regression on Order Statistics [20] was applied. Later, a Principal Component Analysis (PCA) was applied, with the aim of clustering the phenolic compounds in the extracts, according to their different behavior during storage. Finally, a non-parametric analysis of variance with repeated measures was applied for each compound [21], in order to determine which factor was more determinant on the degradation of each phenolic compound. Data analysis was performed with the packages implemented in the software R.3.2.0 (R core team, Free Software Foundation, Boston, MA, USA).

## 3. Results and Discussion

### 3.1. Antioxidant Capacity and Total Phenolic Content

Table 1 shows descriptive statistics (mean ± standard deviation) for the antioxidant capacity and total polyphenol content of grape stem extracts at the beginning of the study and its evolution over 2, 4 and 6 months of storage, under the different conservation conditions. A non-parametric ANOVA with repeated measures was carried out (see Tables S1 and S2, Figures S1 and S2 in the Supplementary Material), to check if tendencies observed in Table 1 were significant. Results of the statistical analysis showed that the antioxidant capacity of the extracts decreased by around 30% and, subsequently, it was not appreciably changed. The antioxidant capacity of samples stored at 25 °C was significantly larger than that of the samples stored at 40 °C (Table S1). Finally, light effect and interactions were not significant.

The total polyphenol content in Mazuelo stem extracts was similar to that observed in grape stem extracts from other varieties such as Merlot [5]. During the storage of the extracts under different conditions, it was observed that the content of total polyphenols decreased significantly over time, especially after six months of storage. Samples stored at higher temperatures showed significant lower polyphenol content values. Finally, light effect and interactions were not significant (Table S2). There is no information on the stability of the total polyphenols from grape stem extracts under different storage conditions. Nevertheless, stability studies of phenolic compounds in other type of extracts have been found. Rocha-Parra et al. [14] analyzed encapsulated wine powder extract stored for 145 days at 38 °C and protected from light, and did not observe significant variations in the total polyphenol content.

### 3.2. Stability of Phenolic Compounds during Storage under Different Conditions of Light and Temperature

Several compounds belonging to different polyphenol families were identified in the grape stem extracts: phenolic acids (gallic acid), flavonoids (catechin, quercetin, quercetin-derived compound, malvidin-3-glucoside, unknown anthocyanin) and stilbenes (resveratrol, viniferin). The antioxidants initially found in the highest concentration in the extracts were the quercetin-derived compounds, catechin and viniferin [8].

Next, the behavior of each phenolic compound during the storage under the previously described conservation conditions will be presented.

#### 3.2.1. Phenolic Acids

**Figure 1:**
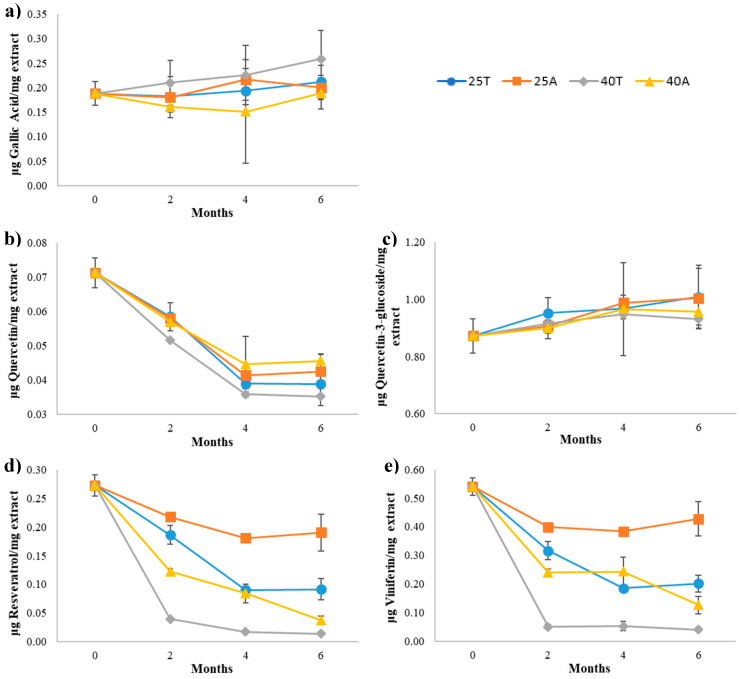
Concentration of gallic acid (**a**), quercetin (**b**), a quercetin derivative (**c**), resveratrol (**d**) and viniferin (**e**) during storage under different conservation conditions over 6 months. Here, 25T, samples conserved at 25 °C in transparent vials; 25A, samples conserved at 25 °C in amber vials; 40T, samples conserved at 40 °C in transparent vials; 40A, samples conserved at 40 °C in amber vials.

As can be seen in Figure 1a, the concentration of gallic acid hardly varied in all the extracts during storage under different conditions. Therefore, this antioxidant was quite stable in grape stem extracts, and was not affected by the light or temperature conditions tested in this study.

Martins et al. [22] concluded that a W1/O/W2 emulsion was required for a gallic acid standard, to maintain more than 50% of its antioxidant capacity for 28 days. However, gallic acid was one of the most stable phenolic compounds in Cabernet Sauvignon wine powder, preserved at 38 °C, and protected from light for 5 months of storage [14]. Galmarini et al. [9] observed that the gallic acid content in freeze-dried and subsequently encapsulated Cabernet Sauvignon wine, remained constant when it was stored in opaque bottles at temperatures of 28 and 38 °C for 70 days of storage. Consequently, it seems that gallic acid stability is greatly influenced by the state it is in (solid or dissolved), as well as the presence or not of other antioxidants jointly.

#### 3.2.2. Flavonoids

Catechin was completely degraded after the first 2 months of storage and, subsequently, it was not detected in any of the samples. Consequently, this compound showed very low stability in grape stem extracts. Flavonoids can undergo oxidation, epimerization, hydrolysis and polymerization when subjected to high temperatures [23]. Rocha-Parra et al. [14] also reported that catechin was one of the most unstable phenolic compounds in an encapsulated wine extract. However, the percentage of loss observed by these authors was 65% instead of >90%, as in our case. This difference may be due to both the protective effect of the encapsulation and the different chemical composition of the extracts. Albuquerque et al. [16] studied the stability of flavan-3-ols in *Arbutus unedo* L. extracts at different temperatures and pHs over a month. They found high degradation rates of these compounds (approximately between 40–60% in most cases), largely influenced by pH and temperature. Despite the different composition between their extracts and ours, the high losses of flavan-3-ols that these authors observed after one month of storage are consistent with the disappearance of catechin in our grape stem extract after two months of storage in different conditions.

Regarding malvidin-3-glucoside, its concentration showed a marked decrease during the first two months of storage under all the conditions studied (Table 2). The most significant decrease was observed in the extracts preserved at 40 °C and exposed to light (40T), while the degradation in the rest of the extracts was similar, independently of the conservation conditions. During the following months, this compound continued to be degraded, until practically disappearing in all the samples. The behavior of the unknown anthocyanin was similar to that of malvidin-3-glucoside, although its degradation was somewhat slower (Table 2). After 4 months of storage, this anthocyanin was detected in all the extracts, although only trace amounts were found in the extracts stored at 40 °C in transparent vials. In the rest of the storage conditions, the degradation of this anthocyanin continued progressively until six months of storage, when it was quantified only in the extracts kept in the most favorable conditions for their conservation (25 °C and protected from light).

Anthocyanins are among the flavonoids that are least stable against temperature [24]. Likewise, other factors, such as pH, light, presence of oxygen, metal ions, enzymes and sugars have also been described as relevant to their stability [25]. Due to the high number of factors that can affect the stability of these compounds, the degradation mechanisms of anthocyanins are not exactly known. However, it is well known that their degradation rate increases with temperature, since it facilitates the shift of the equilibrium towards the chalcone forms [25] and its subsequent irreversible degradation towards colorless simple phenolic forms [26]. Recently, Dangles and Fenger [24] have reported that, in view of the structure of anthocyanin breakdown products, disruption of C2-C1’, C2-C3 and C3-C4 bonds could occur, due to a combination of hydrolytic and autoxidative mechanisms.

**Figure 2:**
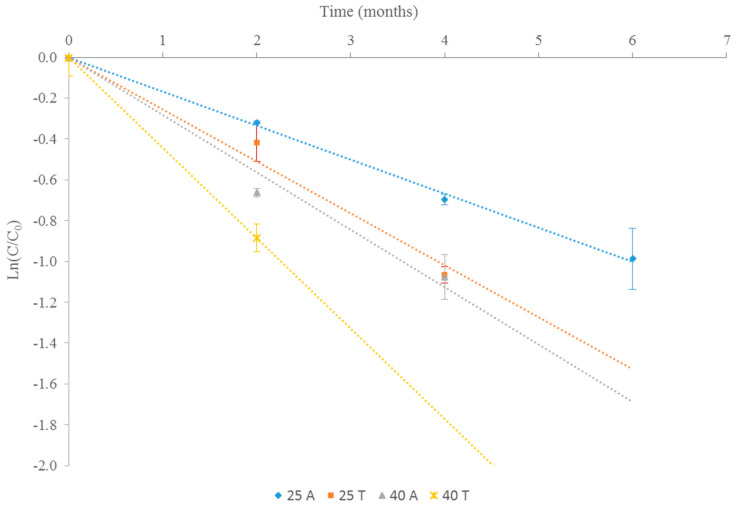
Anthocyanin degradation kinetic at different conservation conditions: ◆: 25 °C in amber vial (25A); ■: 25 °C in transparent vial (25T); ▲: 40 °C in amber vial (40A); ×: 40 °C in transparent vial (40T).

Several studies have found that anthocyanin degradation follows first-order kinetics [27,28]. In the present work, as can be seen in Figure 2, the quantifiable concentration values of this unknown anthocyanin over time also adjusted to a first order kinetics in all cases.

The kinetic parameters for the degradation of this anthocyanin showed increasing values of the kinetic constant and consequently decreasing half-life (t_1/2_) values, as the extracts increase in temperature and light exposure (Table 3). Likewise, it should be noted that both the kinetic constant and the half-life of this compound in the samples stored at 25 °C and exposed to light were very similar to those of anthocyanin in the extracts stored at 40 °C and protected from light. Thus, in this case, the effect of light at room temperature was the same as that of a temperature of 40 °C, but with protection against light.

These values of kinetic constants are generally lower than those described by other authors for anthocyanins subjected to thermal degradation processes [28,29]. This could be due to the fact that, in most of the works found, the stability of these compounds was determined in liquid media and at higher temperatures, and anthocyanins are more susceptible to degradation in solvated state than in powder. However, when samples were stored in powder, kinetic parameters were in the range of values obtained in the present study. For example, Ferrari et al. [30] studied the stability of anthocyanins in an extract of blackberry encapsulated and preserved for 5 months at 25 °C and 35 °C. These authors also observed a first-order kinetic model in the degradation of these compounds, and that temperature negatively influenced their stability. The values of the kinetic constants obtained by these authors (0.057–0.096 month^−1^) were lower than those found in the present study. In fact, half-life values ranged from 7 to 13 months, which means that these values were between 2 and 8 times higher than those found in our study. This difference may be due to the protective effect of maltodextrin and gum arabic used as encapsulating agents, since they are capable of creating a waterproof oxygen wall system that improves anthocyanins stability [30]. Meanwhile, Zorić et al. [31] analyzed the stability of anthocyanins in a cherry extract, encapsulated and stored at 4, 20 and 37 °C for 12 months. In this case, the anthocyanins were the phenolic compounds of the extract that were more easily degraded during storage, with half-life values similar or higher than those found in the present study.

Quercetin concentration decreased progressively in all samples during the first 4 months of storage, and then remained constant (Figure 1b). The degradation was somewhat higher in the samples kept at 40 °C in transparent vials, while in the rest of the samples, was very similar. This behavior suggests that temperature was not the most determining factor in its degradation, and there was some influence of light, mainly when higher conservation temperatures were applied. Holzschuh et al. [32] analyzed the stability of a powdered extract from the medicinal plant *Achyrocline satureioide*, and observed that quercetin concentration increased after three months of storage at 50 °C, in both amber and clear vials. These authors explained this increase due to the possible conversion of an unknown compound into quercetin, since they observed a decrease in that unknown compound. This process could also explain the variation in the degradation trend of this compound observed between months four and six of this study, which prevents data adjustment to a degradation kinetics of order 0, 1 or 2. On the other hand, the quercetin-derivative remained practically stable throughout the six months of storage, regardless of temperature and light conditions applied (Figure 1c). This quercetin derivative was quantified as quercetin-3-glucoside, but the chromatographic technique used did not allow us to distinguish between it and quercetin-3-glucuronide, since both had very similar spectra and retention times [8]. Both structures and, to the best of our knowledge, all the quercetin derivatives described in the literature that have been found in grape stem extracts, correspond to glycosylated derivatives of quercetin in position 3. Considering its structure and the experimental evidence found by different authors, quercetin in its aglycone form has greater antioxidant potential than its derivatives obtained by the derivatization of hydroxyl groups [33]. Beside other structural factors, the presence of hydroxyl groups at positions 3 and 5 involves an increase in the antioxidant potential of this type of compounds [34]. In fact, to maximize free radical neutralization efficiency, the 3-OH group attached to the 2,3-double bond and adjacent to the 4-carbonyl group in the C ring is required [35]. This structural difference could justify the differences in stability found between both compounds. Thus, it seems logical to expect a greater tendency to oxidation and, therefore, to the degradation of quercetin versus its derivative, as was observed in the present study.

#### 3.2.3. Stilbenes

Resveratrol and viniferin showed very similar behavior during storage in all cases (Figure 1d,e, respectively). After two months of storage, decreases in the concentration of both stilbenes were observed, with a clear effect of both temperature and light. The storage conditions that allowed better conservation of these compounds in the extracts were 25 °C in amber vial, preserving approximately 70% of resveratrol and 80% of viniferin after six months of storage. It should also be noted that, unlike the rest of the phenolic compounds analyzed, for the same storage temperature, the degradation rate of stilbenes in extracts exposed to light is clearly higher than that of extracts preserved in amber vials. So, although both factors influenced the stilbenes degradation, the effect of light is more decisive than that of temperature. It is well known that resveratrol breaks down chemically during storage, particularly when it is exposed to light, causing the isomerization of *trans*-resveratrol into *cis*-resveratrol [36]. Resveratrol can be decomposed also into two monomers, including phenol and resorcinol. Therefore, the carbon–carbon double bond in resveratrol shows certain instability [12]. There are several works reporting the potential photo and thermodegradation mechanisms of this compound, as well as different degradation products that can be formed [37,38]. Viniferin, a dimer of resveratrol, shows the same transition from *trans* to *cis* conformation, losing its biological activity. However, its photosensitivity has been reported as less pronounced than that of resveratrol [39]. Most of the studies found for solid state resveratrol have been carried out in the short term or applying very extreme conditions, which are not comparable to the conditions applied in this work. Zhao et al. [13] concluded that exposing *trans*-resveratrol for 4 h to UV light induces the formation of *cis*-resveratrol, and this effect was more marked when this compound was in solution than when it was in powder form. Higher levels of the *cis* isomer were found when resveratrol was in the solution, and in addition, two new compounds that were not found when resveratrol was in powder form were formed in solution. On the other hand, Silva et al. [40] performed a thermoanalytical study of resveratrol in solid form. After analyzing the product resulting from the thermal treatment above 380 °C in nitrogen atmosphere using high-resolution mass spectrometry, they identified dihydro-resveratrol and ε-viniferin or δ-viniferin. However, Galmarini et al. [9] observed that the resveratrol content in encapsulated wine powder remained constant when it was stored in opaque bottles, at temperatures of 28 and 38 °C for 70 days. Again, encapsulation could justify the preservation of this compound.

Comparing the evolution of the antioxidant activity and total polyphenol content with the concentration of the individual polyphenols during storage, a different behavior was detected, as the decrease in antioxidant capacity and total polyphenol content over time is not proportional to that of the individual phenolic compounds. This lack of correlation is in agreement with the results obtained with other authors [14,41,42,43] and was attributed by Moser at al. [43] to the formation of new phenolics, with equal or even improved antioxidant activities that compensate the loss of the original phenolics.

### 3.3. Statistical Analysis of the Influence of Light and Temperature on the Stability of Phenolic Compounds in Grape Stem Extracts

To perform the statistical analysis of the data obtained during the storage of grape stem extracts under different environmental conditions, the concentration value obtained for each of the phenolic compounds at different times was divided by the average concentration at the beginning of the experiment. When the concentration of a certain compound was below the LOQ of the technique (trace amounts), a left-censored data completion model was applied. Thus, we eliminated the bias that in certain statistics assumes a zero value for an observation when that value is not, in fact, zero. This is an appropriate method for our case, because there are few data (<50), with a percentage of censored data of 36% and different LOQs according to the compound. In Supplementary Material (Figure S3), the box plots obtained for each phenolic compound after this data transformation are shown.

**Figure 3:**
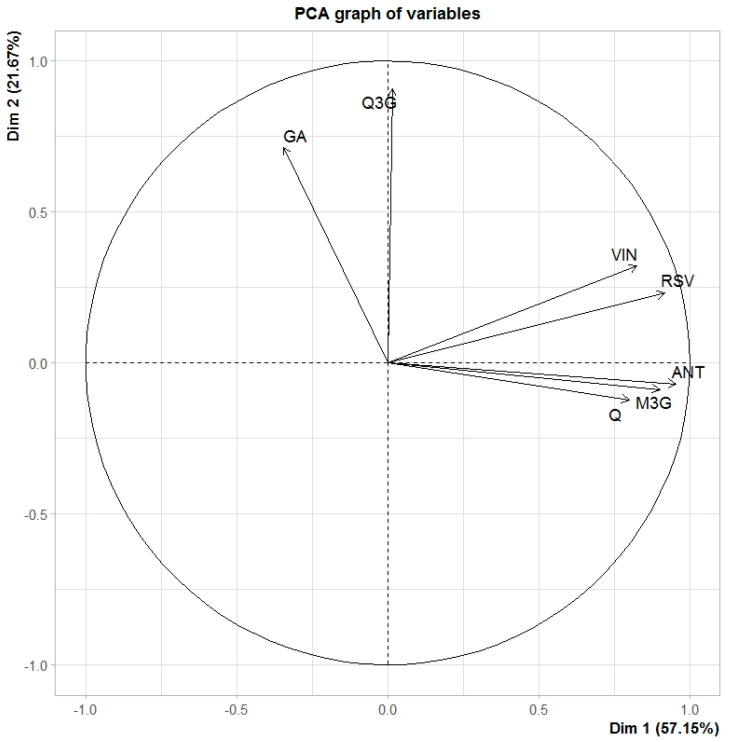
Principal Component Analysis of the following variables: gallic acid (GA), malvidin-3-glucoside (M3G), unknown anthocyanin (ANT), quercetin (Q), quercetin derivative (Q3G), resveratrol (RSV) and viniferin (VIN).

Next, a PCA was performed, in order to summarize all the information in a single illustrative graph. The PCA explained almost 80% of the variability of the data (Figure 3). As can be seen, PCA grouped the variables under study into different clusters, depending on their behavior during the storage. Gallic acid and the quercetin derivative formed the cluster of compounds that were not affected by light and temperature during 6 months of storage. Resveratrol and viniferin are then found clockwise. Both stilbenes were the phenolic compounds that were degraded by both the action of temperature and light. These compounds were the only ones in which the effect of light on their degradation was clearly appreciated. Finally, the third cluster gathers the phenolic compounds with a slightly more complex behavior. In general, anthocyanins were more influenced by the storage temperature than by light, although in the case of the unknown anthocyanin, light did have an appreciable effect, especially in extracts kept at 40 °C. However, in the case of quercetin, it seems that light had more influence than temperature, since the extracts preserved in amber vials had a slightly higher concentration than the extracts preserved without light protection. Catechin could not be represented in this figure, due to its rapid degradation. However, it is clear that catechin would form a group itself, the group of highly unstable compounds, as it was the only compound that disappeared from all samples in just two months.

Lastly, a non-parametric analysis of variance was performed, with repeated measures for each of the phenolic compounds. The results are found in Tables S3–S8 and Figures S4–S9 of Supplementary Material. They confirmed the clusters in the PCA graph, as well as some previously discussed results. Neither gallic acid (Table S3 and Figure S4) nor the quercetin derivative (Table S4 and Figure S5) were influenced by temperature or light throughout the conservation period. It is worth mentioning that the concentration of these compounds showed slight increments over time. These variations could be attributed to the formation of these compounds as products of degradation of other labile molecules. Both anthocyanins analyzed were sensitive to temperature, but the unidentified one was also influenced by light, especially when higher temperatures were applied (Tables S5 and S6, Figures S6 and S7). The effect of light was significant for quercetin degradation, and it also existed in the interaction between light and temperature (Table S7 and Figure S8). Resveratrol, as a representative compound of stilbenes, had a clear influence of both light and temperature on its degradation (Table S8 and Figure S9). The results of viniferin are not shown because they are almost the same as those of resveratrol.

## 4. Conclusions

In this work, the stability of grape stem extracts under different conditions of light and temperature was analyzed during a period of six months. Regarding the behavior of phenolic compounds, catechin disappeared from all the extracts in just two months of storage, regardless of temperature and light, becoming the most unstable compound of the extract. In contrast, gallic acid and a quercetin derivative were not affected by these factors at any time. Some compounds such as stilbenes require protection from light, in addition to temperature control, to prevent their degradation during storage, and thus maintain their biological activity. However, for the conservation of other compounds, such as anthocyanins, temperature control is much more decisive, since light will only affect them appreciably when they are exposed to high temperatures (enhancing effect). Otherwise, the antioxidant capacity of the extracts only decreased about 30% after two months of storage, and subsequently did not vary. Therefore, this would indicate that the degradation products formed from the most unstable phenolic compounds probably also provide some antioxidant capacity to grape stem extracts. Consequently, for the conservation of grape stem extract over time, both temperature control and protection from light are imperatively necessary, in order to increase its shelf life while maintaining its biological properties.

## Figures and Tables

**Table 1:** Antioxidant capacity (mmol Trolox/g grape stem extract) and total phenolic content (mmol gallic acid/g grape stem extract) of grape stem extracts during storage at different conditions of temperature and light (T: transparent vial, A: amber vial).

Time (Months)	Storage Conditions		Antioxidant Capacity	Total Phenolic Content
	T (°C)	Vial		
**0**			0.45 ± 0.05	0.50 ± 0.01
2	25	A	0.33 ± 0.01	0.42 ± 0.01
		T	0.33 ± 0.01	0.44 ± 0.01
	40	A	0.32 ± 0.01	0.40 ± 0.01
		T	0.32 ± 0.01	0.40 ± 0.01
4	25	A	0.31 ± 0.02	0.44 ± 0.02
		T	0.32 ± 0.01	0.43 ± 0.01
	40	A	0.31 ± 0.01	0.42 ± 0.01
		T	0.29 ± 0.02	0.41 ± 0.01
6	25	A	0.33 ± 0.04	0.38 ± 0.06
		T	0.34 ± 0.03	0.38 ± 0.05
	40	A	0.32 ± 0.03	0.36 ± 0.02
		T	0.29 ± 0.01	0.33 ± 0.02

**Table 2:** Concentration of anthocyanins (µg/mg extract) in grape stem extracts during the storage at different conditions of temperature and light (T: transparent vial, A: amber vial).

Time (Months)	Storage Conditions		Malvidin-3-Glucoside	Unknown Anthocyanin
	T (°C)	Vial		
**0**			0.13 ± 0.01	0.14 ± 0.01
2	25	A	0.09 ± 0.01	0.098 ± 0.001
		T	0.08 ± 0.01	0.09 ± 0.01
	40	A	0.07 ± 0.01	0.070 ± 0.001
		T	tr	0.056 ± 0.004
4	25	A	tr	0.067 ± 0.002
		T	tr	0.047 ± 0.002
	40	A	tr	0.046 ± 0.005
		T	nd	tr
6	25	A	tr	0.05 ± 0.01
		T	tr	tr
	40	A	nd	tr
		T	nd	tr

tr = traces.

**Table 3:** Kinetic parameters for the unknown anthocyanin degradation in grape stem extracts stored under different conditions.

Storage Conditions	k (Month^−1^)	t_1/2_ (Month)	R^2^
25 °C, protected from light	0.167 ± 0.024	4.1 ± 0.6	0.997
25 °C, exposed to light	0.255 ± 0.037	2.7 ± 0.4	0.981
40 °C, protected from light	0.281 ± 0.039	2.6 ± 0.4	0.979
40 °C, exposed to light	0.442 ± 0.070	1.6 ± 0.2	1.000 *

* Linear adjustment from only two values. The result obtained in this case should be considered as an estimation.

## Supplementary Materials

The following are available online at https://www.mdpi.com/2076-3921/9/8/720/s1, Table S1: Influence of different factors on the antioxidant capacity of grape stem extracts during storage. Table S2: Influence of different factors on the phenolic content of grape stem extracts during storage. Table S3: Influence of different factors on gallic acid during storage of grape stem extracts. Table S4: Influence of different factors on the quercetin derivative during storage of grape stem extracts. Table S5: Influence of different factors on the unknown anthocyanin during storage of grape stem extracts. Table S6: Influence of different factors on malvidin-3-glucoside during storage of grape stem extracts. Table S7: Influence of different factors on quercetin during storage of grape stem extracts. Table S8: Influence of different factors on resveratrol during storage of grape stem extracts. Figure S1: Plot of means with intervals for antioxidant capacity of grape stem extracts during storage. Figure S2: Plot of means with intervals for total phenolic content of grape stem extracts during storage. Figure S3: Box plot for each phenolic compound after the data transformation applied. Figure S4: Plot of means with intervals for gallic acid during storage of grape stem extracts. Figure S5: Plot of means with intervals for quercetin derivative during storage of grape stem extracts. Figure S6: Plot of means with intervals for the unknown anthocyanin during storage of grape stem extracts. Figure S7: Plot of means with intervals for malvidin-3-glucoside during storage of grape stem extracts. Figure S8: Plot of means with intervals for quercetin during storage of grape stem extracts. Figure S9: Plot of means with intervals for resveratrol during storage of grape stem extracts.
