# Supplementary material for: Stability of Phenolic Compounds in Grape Stem Extracts
Source: Antioxidants (Basel). 2020 Aug 8;9(8):720. doi: 10.3390/antiox9080720 (PMC7464403; doi:10.3390/antiox9080720)
Supplement: Supplementary file 1 [file antioxidants-09-00720-s001.pdf]

Supplementary Material

Figure S1. Box plot for each phenolic compound after the data transformation applied

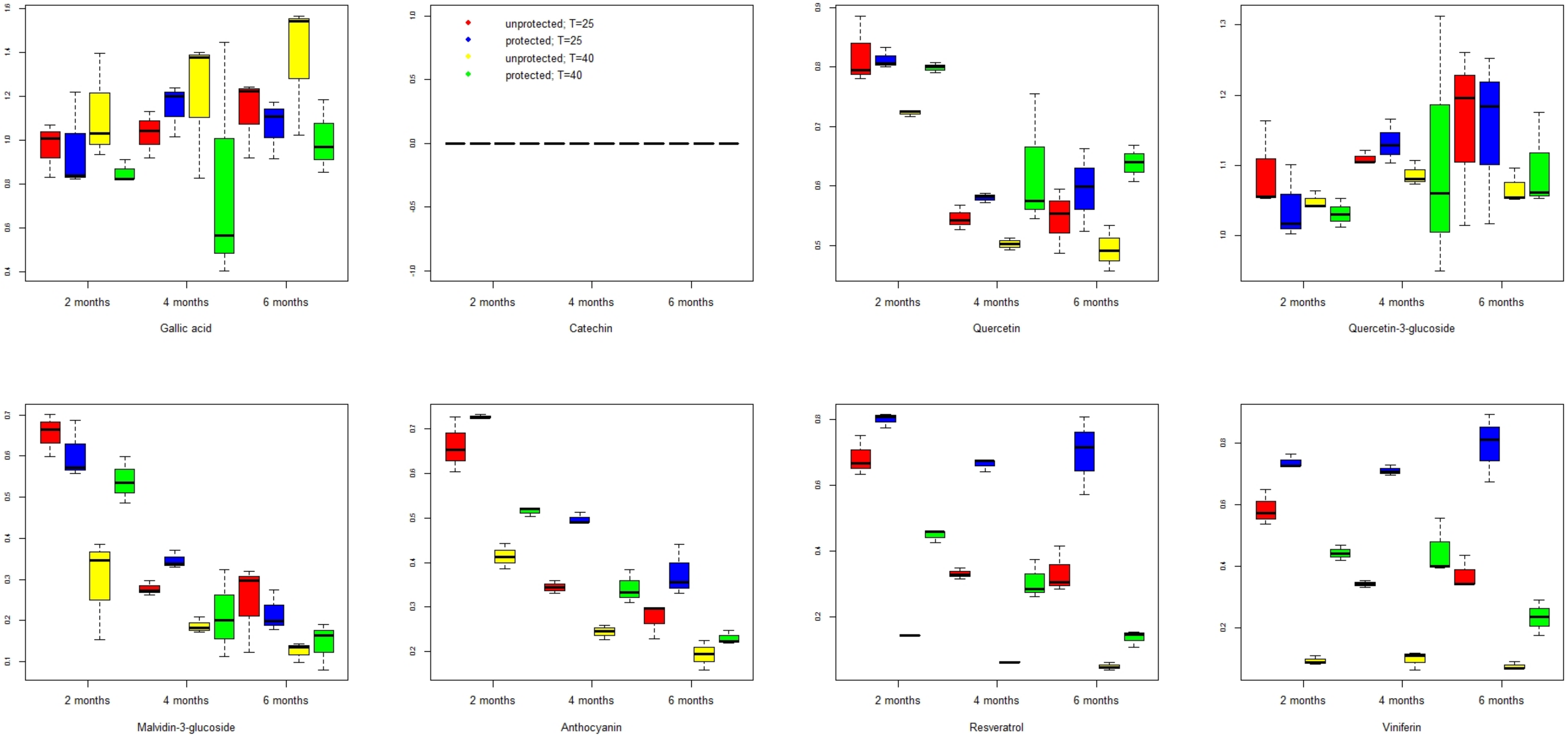

Non-parametric analysis of variance with repeated measures

A non-parametric analysis of variance with repeated measures was applied for each phenolic compound. Observe that we can distinguish two kind of factors, first, Light and Temperature which are global factors (between subjects) and create independent observations for each combination of levels and, second, the factor Time that distinguishes observations on the same individual (within subjects) at different time points and creates correlated observations. After the analysis we obtained three kind of results that confirm previous discussion (Tables S1-S6 and Figures S2-S7). We confirmed previous discussion and the groups formed in the component analysis. Gallic acid and the quercetin derivative do not present differences between global factors, and only a different level over time is significant. Resveratrol and viniferin give almost the same results (therefore, viniferin results are not shown) and clearly have different behavior for treatments in Light, and Temperature. The unknown anthocyanin has different behavior for treatments in Temperature, and Light interacts with time. Finally, malvidin-3-glucoside had different behavior depending on Light but not on Temperature and quercetin showed interaction between L and T. This flavonoid was the only compound, where protection makes larger values with high temperature.

a) Gallic acid (GA)

Table S1. Influence of different factors on the stability of gallic acid during storage of grape stem extracts

Gallic Acid	DF	Statistic
Light (L)	1	1.88
Temperature(T)	1	0.02
Time (t)	2	9.29**
L*T	1	1.82
L*t	2	2.63
T*t	2	1.66
L*T*t	2	1.19

*p<0.05, **p<0.01, ***p<0.001

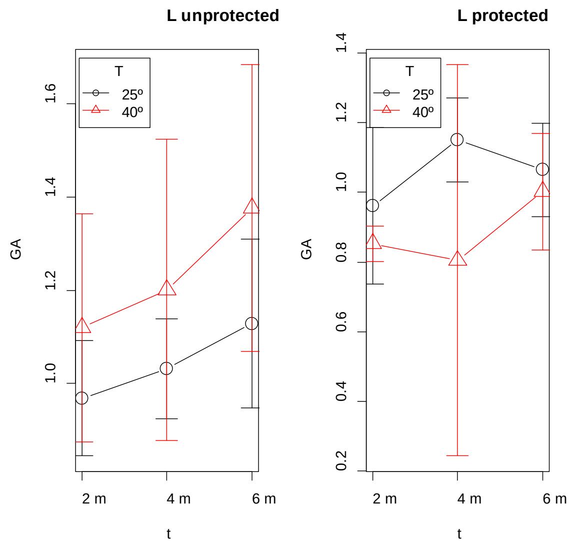

Figure S2. Plot of means with intervals for gallic acid during storage of grape extracts

b) Quercetin derivative (Q3G)

Table S2. Influence of different factors on the stability of quercetin derivative during storage of grape stem extracts

Quercetin Derivative	DF	Statistic
Light (L)	1	0.32
Temperature(T)	1	3.87
Time (t)	2	13.56**
L*T	1	0.05
L*t	2	0.79
T*t	2	0.10
L*T*t	2	1.30

*p<0.05, **p<0.01, ***p<0.001

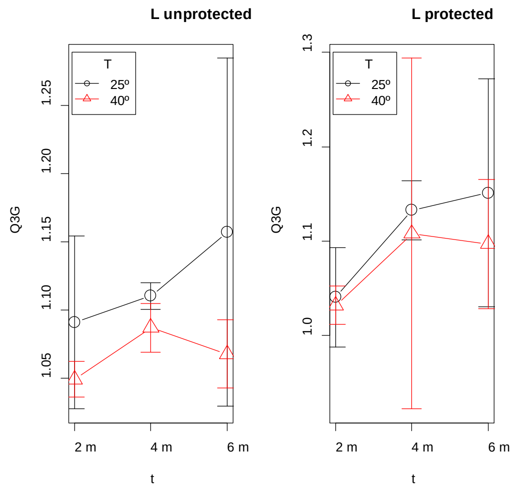

Figure S3. Plot of means with intervals for quercetin derivative during storage of grape extracts

c) Unknown anthocyanin (ANT)

Table S3. Influence of different factors on the stability of the unknown anthocyanin during storage of grape stem extracts

Anthocyanin	DF	Statistic
Light (L)	1	34.57***
Temperature(T)	1	78.73***
Time (t)	2	456.20***
L*T	1	0.10
L*t	2	9.05**
T*t	2	1.31
L*T*t	2	7.29**

*p<0.05, **p<0.01, ***p<0.001

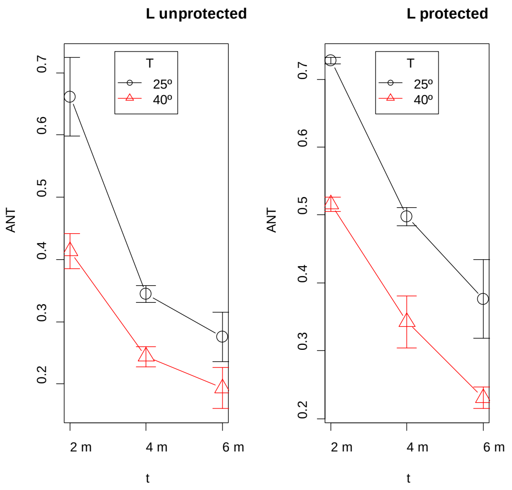

Figure S4. Plot of means with intervals for the unknown anthocyanin during storage of grape extracts

d) Malvidin-3-glucoside (M3G)

Table S4. Influence of different factors on the stability of malvidin-3-glucoside during storage of grape stem extracts

**Malvidin-3-glucoside**	**DF**	**Statistic**
Light (L)	1	1.69
Temperature(T)	1	14.83**
Time (t)	2	53.78***
L*T	1	1.82
L*t	2	1.34
T*t	2	0.01
L*T*t	2	8.68

*p<0.05, **p<0.01, ***p<0.001

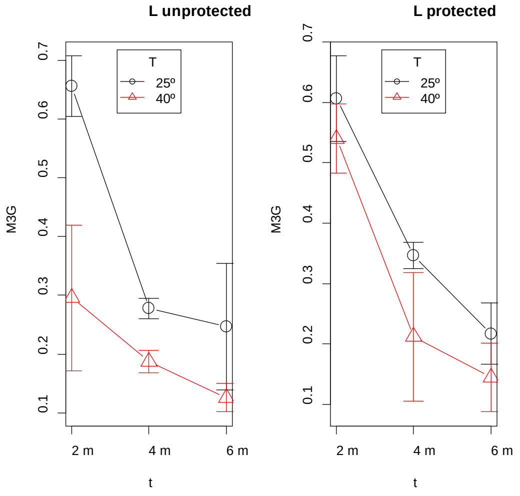

Figure S5. Plot of means with intervals for malvidin-3-glucoside during storage of grape extracts

e) Quercetin (Q)

Table S5. Influence of different factors on the stability of quercetin during storage of grape stem extracts

Quercetin	DF	Statistic
Light (L)	1	35.17***
Temperature(T)	1	2.38
Time (t)	2	110.10***
L*T	1	8.09***
L*t	2	2.49
T*t	2	0.68
L*T*t	2	0.61

*p<0.05, **p<0.01, ***p<0.001

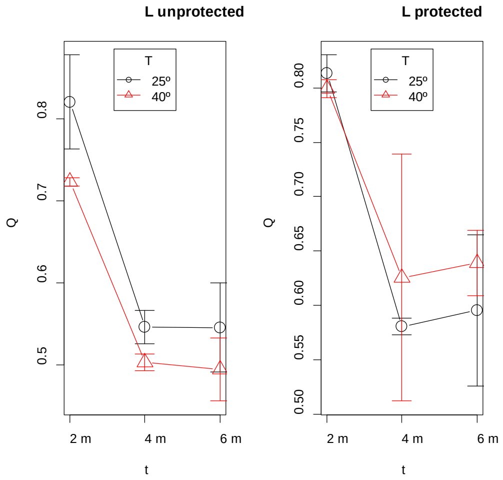

Figure S6. Plot of means with intervals for quercetin during storage of grape extracts

f) Resveratrol (RSV)

Table S6. Influence of different factors on the stability of resveratrol during storage of grape stem extracts

Resveratrol	DF	Statistic
Light (L)	1	144.75***
Temperature(T)	1	323.36***
Time (t)	2	104.98***
L*T	1	0.42
L*t	2	0.55
T*t	2	9.63**
L*T*t	2	8.71**

*p<0.05, **p<0.01, ***p<0.001

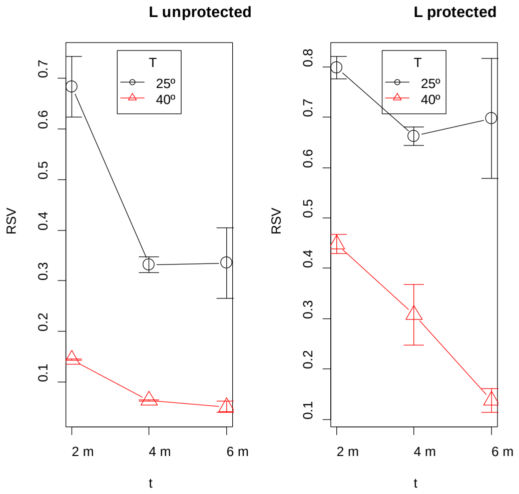

Figure S7. Plot of means with intervals for resveratrol during storage of grape extracts
